# Bovine Brucellosis in Gauteng, South Africa: Seroprevalence amongst Cattle Handlers and Variables Associated with Seropositive Cattle Herds, 2014–2016

**DOI:** 10.3390/pathogens10121547

**Published:** 2021-11-26

**Authors:** Krpasha Govindasamy, Peter N. Thompson, Bernice N. Harris, Jennifer Rossouw, Darrell A. Abernethy, Eric M. C. Etter

**Affiliations:** 1 Department of Production Animal Studies, Faculty of Veterinary Science, University of Pretoria, Onderstepoort 0110, South Africa; peter.thompson@up.ac.za (P.N.T.); eric.etter@cirad.fr (E.M.C.E.); 2 School of Health Systems and Public Health, University of Pretoria, Pretoria 0031, South Africa; bernice.harris@up.ac.za; 3 Centre for Emerging Zoonotic and Parasitic Diseases, National Institute for Communicable Diseases, Johannesburg 2192, South Africa; jennyr@nicd.ac.za; 4 Centre for Veterinary Wildlife Studies, Faculty of Veterinary Science, University of Pretoria, Onderstepoort 0110, South Africa; daa47@aber.ac.uk; 5 Aberystwyth School of Veterinary Science, Institute of Biological, Environmental and Rural Sciences, Aberystwyth University, Penglais, Aberystwyth SY23 3FL, UK; 6 CIRAD, UMR AnimalS Health Territories Risks Ecosystems (ASTRE), 34070 Montpellier, France; 7 ASTRE, Univ Montpellier, CIRAD, INRA, 34070 Montpellier, France

**Keywords:** brucellosis, cattle handler, veterinary official, seroprevalence, BrucellaCapt^®^, IgG ELISA^®^, IgM ELISA^®^, RBT^®^, *B. abortus*, South Africa, risk factor

## Abstract

In South Africa, the prevalence of cattle handler exposure to *Brucella* on cattle farms is unknown and risk factors and cattle symptoms associated with infected cattle herds are unavailable. To address this gap, a case-control study of cattle herds was conducted in Gauteng province and farm workers and veterinary officials were tested for exposure to *Brucella*. Seroprevalence amongst farm workers exposed to case herds ranged from 4.0% (BrucellaCapt^®^) to 16.7% (IgG ELISA^®^), compared to those exposed to control herds, where seroprevalence ranged from 1.9% (BrucellaCapt^®^) to 5.7% (IgG ELISA^®^). Seroprevalence amongst veterinary officials was significantly greater compared to farm workers exposed to case herds for the outcome RBT+ IgM- IgG+ (OR = 11.1, 95% CI: 2.5–49.9, *p* = 0.002) and RBT- IgM- IgG+ (OR = 6.3, 95% CI: 2.3–17.3, *p* < 0.001). Risk factors associated with being an infected herd were: being a government-sponsored farm vs. private farm (OR 4.0; 95% CI: 1.4–11.3; *p* = 0.009), beef vs. dairy herd (OR 7.9; 95% CI: 1.4–44.9; *p* = 0.020), open vs. closed herd (OR 3.3; 95% CI: 1.1–10.4; *p* = 0.038) and the presence of antelope on the farm (OR 29.4; 95% CI: 4.0–218.2; *p* = 0.001). Abortions (OR = 5.1; 95% CI: 2.0–13.3; *p* < 0.001), weak calves in the herd (OR = 8.0; 95% CI: 2.6–24.4; *p* < 0.001), reduction in number of calves born (OR = 9.0; 95% CI: 2.1–43.6; *p* < 0.001), reduction in conception rate (OR = 3.9; 95% CI: 0.8–18.3; *p* = 0.046), hygromas in cattle (*p* = 0.011) and farmers reporting brucellosis-like symptoms in their farm workers or in him/herself (OR = 3.4; 95% CI: 1.3–8.7; *p* = 0.006) were more likely to be associated with *Brucella* infected herds than control herds. This evidence can be used in strategic planning to protect both human and herd health.

## 1. Introduction

Brucellosis is a neglected zoonotic bacterial disease impacting public health and global agricultural development [1,2,3,4]. *Brucella abortus* causes bovine brucellosis [5,6] and can be transmitted directly or indirectly to people through contact with uterine discharges of infected animals or the ingestion of unpasteurised dairy products. The preferred host is cattle, but it may also occur in wildlife species, such as eland and impala [7,8]. To date, the most effective method to prevent human brucellosis is to eliminate the infection from livestock [6,9].

Having one or more of the following symptoms is characteristic of cattle infected with brucellosis: abortion, retained placenta, stillbirths, poor weight gain, orchitis, epididymitis and hygromas [10]. Abortion caused by *B. abortus* in cattle usually occurs in the third trimester due to necrotising placentitis [6] and exposure to these tissues is the primary source of transmission to humans or uninfected bovine, which occurs through aerosolized or direct mucosal contact [11]. However, infection in cattle does not always lead to abortion, but can persist in a herd without any overt clinical symptoms, other than the birth of weak or nonviable calves and a reduction in milk yield [11,12]. Localisation of the bacterium occurs in male reproductive tissue, joints and bones, and within the mammary glands, resulting in sterility, hygromas and mastitis, respectively [11]. Infection spread by bulls during natural service is reported to be rare [11,13]. Other infection sources include contaminated environments, especially if it is wet and muddy, and equipment used for milking or artificial insemination [10]. In utero infection or milk and colostrum can also be sources of disease transmission to the new-born calf [13].

Since some symptoms of bovine brucellosis are covert, once the disease has established itself in the herd it is difficult to rapidly detect and, therefore, difficult to control [12,14]. Two main factors contribute to this situation. Firstly, the disease has a highly variable incubation period of several months to at least 2 years, and up to 9 years [10,15]. Secondly, the host’s immunological response affects detection of the disease, with 2.5–9% of infected heifers born from seropositive cows remaining seronegative on conventional serological tests for at least 18 months [10,16]. These challenges necessitate extended surveillance and control activities to eliminate brucellosis from a herd [17]. It takes a minimum of two years after the documented absence of reactors [18,19] to declare a herd free, and may take several decades to declare a country free from brucellosis. The duration of successful eradication programs varies greatly between countries, ranging from 23 years in New Zealand and 29 years in Australia [20] up to 100 years in Malta [21].

Symptoms of brucellosis in humans are just as non-specific as in animals. However, unlike abortions in the cattle herd, the main symptom of acute infection in humans is a recurring febrile illness, difficult to distinguish from other febrile illnesses [22]. Other symptoms include malaise, anorexia, muscular weakness, joint pain, back pain and depression. The disease can also result in bone and testicular abscesses, endocarditis, and neurological complications [23]. Persons suffering from infection of a long evolution (“chronic”) are reported to experience chronic disability and time lost from daily activities [23]. There is no vaccine against the disease for humans [5], and successful treatment of the disease depends on early detection and initiation of the correct combination of antibiotics [23,24].

For similar biological reasons, detecting brucellosis in humans is as tricky as detecting the disease in cattle. Al Dahouk et al., (2011) reviewed the difficulties in diagnosis of human brucellosis through culture and molecular methods which justify the use of serological tests [5,25,26]. However, these authors also point out the difficulties in clinical interpretation of serological test results in patients living in *Brucella* endemic areas.

In addition to the diagnostic challenge of brucellosis driving the neglect of the disease, a paucity of recent quantitative evidence confirming the interrelationship between the prevalence of the animal disease and human disease, contributes to decreasing prioritization of the disease by government and policymakers [27,28]. Previously, it was accepted that the incidence of human brucellosis correlates with the incidence of brucellosis in livestock [29,30]. However, more recent reviews recognize that this may not always be the case and can depend on multiple variables, including proximity to the herd and eating and cultural habits [22].

Quantitative evidence of human exposure to *Brucella* spp. linked to seropositive cattle is possible to attain by conducting integrated epidemiological studies on animal and human brucellosis. However, these studies are difficult mainly because zoonotic disease detection in humans and animal hosts are separate and siloed [31,32,33,34]. As a result, brucellosis data for humans and animals are usually presented separately and not epidemiologically linked by time or location [35], or datasets are incomplete because only animal or human data are available [36,37,38].

In South Africa (SA), bovine brucellosis is a controlled animal disease and human brucellosis a notifiable medical condition, however despite the zoonotic nature of *Brucella*, to date there is no published record of a multidisciplinary epidemiologic study of brucellosis, conducted by veterinary officials in collaboration with medical doctors. In addition, despite a known long history of bovine brucellosis in Gauteng province [39], no investigation has been undertaken to determine herd management risk factors or cattle symptoms associated with seropositive cattle herds.

This study therefore aimed to measure human exposure to *Brucella* on cattle farms participating in the bovine brucellosis control programme of Gauteng Veterinary Services to firstly understand the evolution of infection amongst farm workers and veterinary officials exposed to *Brucella*-infected cattle herds and to determine herd management risk factors and cattle symptoms associated with herd-level infection in the province.

## 2. Results

In total, 133 cattle herds were recruited into the study, of which 30 met the definition of a case farm and 103 were classified as control farms. The average herd size on case farms and control farms was 196 (median: 120; IQR: 71–238) and 150 (median: 100; IQR: 43–218) cattle.

### 2.1. Human Exposure to Brucella and Evolution of Infection

**Figure 1:**
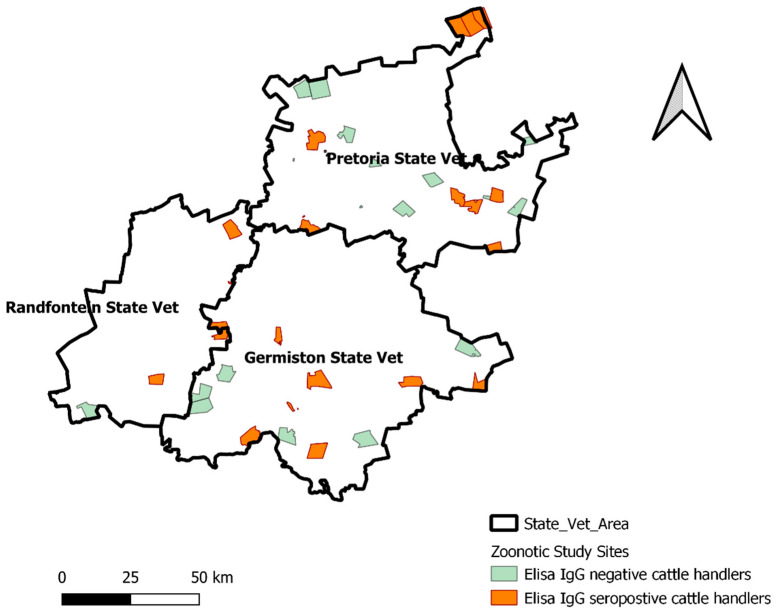
Distribution of farm parcels included in the study with *Brucella* IgG ELISA seropositive and negative farm workers, Gauteng, 2016.

In total, 230 individuals were tested, ranging in age from 16 to 75 (median: 38; IQR: 32–49). In this study, the median number of workers per farm was four persons (IQR: 3–7; range: 1–16). Figure 1 illustrates the spatial dispersion of *Brucella* IgG ELISA^®^ seropositive farm workers across the province.

Seroprevalence amongst farm workers on case farms (*n* = 30 farms) ranged from 4.0% (BrucellaCapt^®^) to 16.7% (IgG ELISA^®^), compared to control farms (*n* = 11 farms), where this seroprevalence ranged from 1.9% (BrucellaCapt^®^) to 5.7% (IgG ELISA^®^) (Table 1).

Overall, 5.7% (13/230) of persons tested were seropositive to the RBT^®^ and IgM ELISA^®^ and IgG ELISA^®^ tests and 3.9% (9/230) were seropositive to all four serological tests. Farm workers on control farms presented with infection of short to longer evolution, compared to the short to very long infection evolution present amongst farm workers on case farms (Table 2).

The difference in seroprevalence amongst farm workers between case and control farms for all the test combinations was not significant. However, seroprevalence amongst veterinary officials was significantly greater compared to farm workers on case farms for the RBT+ IgM- IgG+ outcome (OR = 11.1, 95% CI: 2.5–49.9, *p* = 0.002) and for the RBT- IgM- IgG+ outcome (OR = 6.3, 95% CI: 2.3–17.3, *p* < 0.001).

### 2.2. Univariate and Multivariable Analysis of Risk Factors for Case Herds

Open herd management was identified as significant on the univariate analysis (*p* = 0.032). However, *Brucella* testing of cattle before introduction into the herd and vaccination (RB51) of cattle introduced into the herd were both not significant (*p* = 1.0). Open herd management and several factors associated (*p* < 0.2) with herd *Brucella* infection status in the univariate analysis (Table 3) were selected for inclusion in the multivariable model.

In the final model (Table 3), being a government-sponsored farm (OR 4.0; 95% CI: 1.4–11.3; *p* = 0.009), beef vs. dairy herd (OR 7.9; 95% CI: 1.4–44.9; *p* = 0.020), open vs. closed herd (OR 3.3; 95% CI: 1.1–10.4; *p* = 0.038) and the presence of antelope on the farm (OR 29.4; 95% CI: 4.0–218.2; *p* = 0.001) were significantly associated with herd *Brucella* infection status. The Hosmer–Lemeshow goodness of fit test indicated adequate fit (χ^2^ = 14.0, *p* = 0.300).

### 2.3. Univariate Analysis of Cattle and Herd Symptoms of Bovine Brucellosis

In the univariate analysis of cattle and herd symptoms (Table 4), abortions (OR = 5.1; 95% CI: 2.0–13.3; *p* < 0.001), weak calves in the herd (OR = 8.0; 95% CI: 2.6–24.4; *p* < 0.001), reduction in number of calves born (OR = 9.0; 95% CI: 2.1–43.6; *p* < 0.001), a reduction in conception rate (OR = 3.9; 95% CI: 0.8–18.3; *p* = 0.046) and hygromas in cattle (*p* = 0.011) were more likely to have been reported by farmers of *Brucella* infected herds than those on in control farms.

In addition to these cattle and herd symptoms, farmers of case herds were significantly more likely to report brucellosis-like symptoms having occurred in farm workers on the farm or in him/herself (OR = 3.4; 95% CI: 1.3–8.7; *p* = 0.006).

## 3. Discussion

This study presents new evidence of cattle handler and veterinary official exposure to *Brucella* on cattle farms participating in the bovine brucellosis control programme of Gauteng, resulting in antibody profiles typical of infection ranging from a short to long evolution. The difference in seroprevalence amongst farm workers between case and control farms for all the test combinations was not significant. However, seroprevalence amongst veterinary officials was significantly greater compared to farm workers on case farms for the RBT+ IgM- IgG+ outcome (*p* = 0.002) and for the RBT- IgM- IgG+ outcome (*p* < 0.001).

*Brucella* seroprevalence in veterinary officials and assistants, has previously been explained by greater exposure to infected reproductive material, accidental exposure to *Brucella* vaccine strains, through needle stick injuries and noncompliance with use of protective clothing [40,41,42]. In our study, all seropositive veterinary officials were animal health technicians (AHTs). The most likely source of exposure for AHTs is accidental needle stick injury during vaccination of cattle herds with S19. Vaccination against and testing of cattle herds for brucellosis are amongst the main activities of AHTs in the province and assistance to veterinarians performing deliveries in cattle is limited. Furthermore, use of protective clothing during sampling and vaccination is sporadic (personal communication, 2016). AHTs will be performing a greater number of vaccinations more frequently than cattle handlers exposed to a single herd. This may explain the difference in seroprevalence between these groups.

In this study, we found that the pattern of *Brucella* antibody expression in the group tested ranged from profiles associated with infection of short evolution, typified by a predominance of IgM, to infection of long evolution in which IgM decreases and IgG (and IgA) increases and eventually predominates over IgM. We also found a class of long evolution categorised by the presence of IgG and low levels of non-agglutinating antibodies (RBT^®^ negative and BrucellaCapt^®^ negative). The antibody profile amongst this group of seropositive farm workers and veterinary officials, indicates that participants were are at different stages in the evolution of infection. It is currently unknown if there are specific risk factors or symptoms significantly associated with these stages in the evolution of infection in this group of people. Further investigation will be needed to clarify this.

Discrepancies between seroprevalence measured using the RBT^®^, IgG ELISA^®^ and BrucellaCapt^®^ test for screening cattle handlers at the human–cattle farm interface is not unexpected, since test sensitivity is associated with the class of circulating antibody at the time of testing [25,26] and is correlated with the cut-off used to distinguish between clinical brucellosis and exposure to *Brucella*. In an endemic area, cut-offs of commercial tests need to be adjusted according to the seroprevalence of *Brucella* exposure in the healthy population [43]. A cut off of 1/320 is recommended for the serum agglutination test in endemic areas [43]. In this study the cut off for the BrucellaCapt^®^ test was 1/320, with reactors below this titre being regarded as negative. Our findings suggest that the IgG ELISA^®^ is not well adjusted to differentiate between low levels of IgG antibody circulating in exposed farm workers and veterinary officials and potential undetected clinical cases of brucellosis in this group. When considering the high sensitivity and specificity of RBT as described in Diaz et al., (2011) and comparing seroprevalence according to the RBT^®^ test with that of the BrucellaCapt^®^ test used in this study, the RBT^®^ when used on its own or in combination with IgM ELISA^®^ or IgG ELISA^®^, was found to be more sensitive than the BrucellaCapt^®^ test. However, no serial dilutions were conducted for the RBT^®^ test in this study as compared to the Diaz et al., (2011) study, which may be indicating that the RBT^®^ is sensitive to titres less than 1:320. The implication being, that if RBT^®^ is to be used in the clinical setting, serial dilutions are recommended, and a suitable cut-off should be determined to differentiate between disease and asymptomatic infection. Findings from this study, however, illustrate that at least 2.2% to 3.9%, if we consider the combination of tests to be most specific, or 6.5%, if we consider only the BrucellaCapt results, of those tested, had titres high enough to be considered clinical cases. The clinical implication is that delayed diagnosis and treatment is associated with increased risk of complicated focal brucellosis [44], treatment failure and relapses [24].

Differences in seroprevalence between cattle handlers and veterinary officials of this study, compared to cattle handlers in other African countries can be explained by differences in exposure due to different herd management systems across countries or study designs. Seroprevalences amongst villagers in Togo and small-scale farmers in Tanzania according to the RBT test were 0.44% and 5.5%, respectively [45,46]. This is lower than seroprevalences in cattle handlers on case farms (8.7%) and control farms (3.8%) found in our study using the RBT test alone (Table 1). In contrast 10.1% of cattle handlers tested in Ghana [47] and 10.4% of farm-workers, abattoir workers and veterinarians in Ethiopia screened using the RBT [22,48] were higher than the seroprevalences found in cattle handlers on both the case and control farms in this study, but still lower than that found in the veterinary officials (29.6%). However, it was equal to the overall prevalence of cattle handlers and veterinarians (10.1% in Table 1)

Two tests were used to test seroprevalence amongst villagers in Togo. Variation of seroprevalence ranged from 0.44% using the RBT alone to 0.73% on the IgG ELISA. The higher seroprevalence amongst cattle handlers, according to the RBT and IgG ELISA found in this study compared to the study conducted in Togo [45] may be explained by the fact that in this study, majority of cattle handlers were exposed to known serologically confirmed *Brucella* seropositive cattle farms. This may be explained by the fact that our study specifically selected cattle handlers exposed to *Brucella* infected herds in contrast to the randomized cross-sectional study design used in the Togo study.

This is the first study in Gauteng province to identify herd management risk factors and cattle symptoms associated with *Brucella* seropositive cattle herds. Furthermore, it is the first study in SA to identify an association between brucellosis-like symptoms in cattle handlers exposed to seropositive cattle herds serviced by the provincial veterinary services.

Beef herds, government funded project herds or herds that were in contact with antelope were associated with *Brucella* infected herds. Government-funded herds being a risk factor suggests that socio-political variables have an indirect effect on herd health, lending credibility to the complex nature of bovine brucellosis control. Such complexity has been discussed in detail by [49,50]. Furthermore, the association found between government-sponsored farms and case herds is not consistent across SA, as illustrated in findings from a recent study conducted in KwaZulu-Natal, South Africa [51], where government-sponsored herds were less likely to be infected. This may be due to the variation in provincial government programmes and in how farms were selected for government funding, or possibly the separation between the agriculture and veterinary state functions in Gauteng, resulting in the distribution of cattle of unknown *Brucella* status to farmers.

The finding of abortions associated with case herds is expected [11,52,53,54] and reported in sub-Saharan Africa [55,56,57,58,59], and South Asia [60,61]. From the Latin America and Caribbean region, abortions were not significantly associated with positive herds in a study [62], which contradicted a study from the same region that found that the RB51 vaccine strain resulted in cattle abortions [63]. A reduction in the number of calves born, is also a commonly reported herd symptom associated with chronic brucellosis in a herd [64]. This is most likely due to the combination of a reduction in conception rate and an increase in abortions in the herd. In a study conducted in Ethiopia, an increase in calving interval was reported to be associated with positive herds [65]. This would also result in the reduction in calves born and may be applicable to the herds investigated in our study. Other reported herd symptoms and risk factors, such as herd size, sex and fenced-in camps [62,66,67], were not found to be significant in our study area.

The reasons for the strong association between the presence of antelope and *Brucella* herd infection are unknown, although it should be interpreted with caution due to the relatively small number of herds with antelope. It is possible that there were other, unmeasured management or environmental factors associated with the presence of antelope which may be related to likelihood of *Brucella* infection. Further investigation is needed to identify the species of antelope most associated with reactor cattle herds in Gauteng. However, the risk of transmission of *B. abortus* between infected wild ungulates and livestock is well documented [68,69], and the presence of a possible wildlife reservoir may hinder efforts at eradication. Findings from this study support the need for further research into the potential role of wildlife in the maintenance of *B. abortus* on cattle farms in Gauteng and elsewhere.

A limitation of the study is that inferences cannot be generalized to the population of cattle handlers or cattle herds beyond those that participated in the provincial bovine brucellosis control programme. Furthermore, since this was a voluntary study, the selected herds and human population investigated reflect the farmers who participated. Only cattle handlers and veterinary officials present on testing day were included. This excludes those cattle handlers that may not have been present due to ill health or other work commitments. Furthermore, the study design did not include follow up testing of seropositive cattle handlers. Therefore, it was not possible to differentiate between asymptomatic infection, active infection, or previous resolved infection. Despite these limitations, the identification of risk factors and herd symptoms of brucellosis and evidence of cattle handler exposure, provides sufficient justification for further pathologic surveillance of both cattle and cattle handlers in the region.

## 4. Materials and Methods

The Research Ethics Committee, Faculty of Health Sciences, University of Pretoria (74/2015) and the Animal Ethics Committee of the Faculty of Veterinary Science, University of Pretoria (V011–16) granted ethical approval for the study.

**Figure 2:**
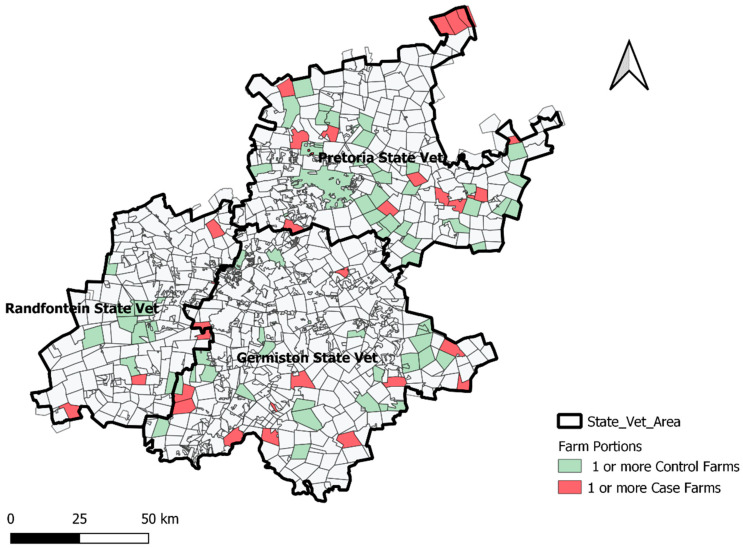
Location of Gauteng province in SA and distribution of study case (*Brucella*-infected) and control cattle herds by farm parcels within state vet areas in Gauteng province, 2014–2016.

This study was conducted in SA’s smallest, but most populated province, Gauteng, which covers 18,176 km^2^. The province is divided into three state veterinary areas, each covering one or more human health districts. Herds are typically clustered into farm parcels within state vet areas. Figure 2 illustrates the spatial distribution of case and control herds participating in this study, within state vet areas. One or more herds can occur within a farm parcel. If both a case and control herd occurred within a farm parcel, the parcel was coded as having one or more case herds. Therefore, farm parcels marked as one or more control herds, had no detected case herds in the parcel.

### 4.1. Selection and Classification of Case and Control Cattle Herds

All herds participating in the provincial veterinary services’ voluntary bovine brucellosis control programme between 2014–2016 were eligible for this study. The bovine brucellosis control programme is a passive surveillance system in which farmers volunteer to have their herds tested. However, if the herd tests positive the farmer must comply with the veterinary regulations to control bovine brucellosis.

After routine veterinary regulatory testing of the herd using the Rose Bengal test (RBT) and confirmation of reactors with the complement fixation test (CFT) [7], farms were categorised as either case or control. Based on the laboratory test records, a cattle herd with two or more serological cattle reactors on the RBT and confirmatory CFT with a reaction of greater than 60 IU/mL, between 2014–2016, was classified as a case herd. The 60 IU/mL threshold for the CFT was selected to rule out the S19 vaccine reactors according to the national veterinary guidelines [18]. The case definition ‘two or more cattle reactors in a herd’ was chosen to increase the specificity of a herd diagnosis of brucellosis and select herds presenting greater risk for cattle handler exposure. A cattle herd with a laboratory-confirmed seronegative test between 2014–2016 and no history of a seropositive herd test during 1990 to 2014, was regarded as a control herd. Verification of case and control classifications was done by cross-checking case herd records, reported by the state veterinarians, with the provincial veterinary services’ animal health directorate in the annual animal health reports. Selection of case and control herds was limited to the period between 2014 and 2016 due to the available budget for testing farm workers on the farm. Farm managers of case herds, identified by state veterinarians and animal health technicians were contacted telephonically and invited to participate in the study until we reached a maximum of 200 farm workers that could be tested. This resulted in an initial sample of 41 case farms. For controls, all available controls that could be contacted in the limited available time (*n* = 92) were included. After verification of the herd status, carried out as described above, 11 of the case herds, were reclassified as control herds, resulting in 30 case herds and a total of 103 control herds. Definitions of selected risk factors that were investigated are shown in Table below (Table 5).

Herd status was used as the response variable for univariate and multivariable analyses conducted to identify herd management factors and symptoms associated with case herds. Herd management risk factors with two categories were tested using the two-sided Fisher test, and the Chi-squared test was used to analyse factors with more than two categories. Variables associated with case herds, at significance *p* < 0.2 in the univariate analyses, were included in a multivariable logistic regression model. Backward stepwise selection was used to identify significant (*p* < 0.05) factors. Model fit was assessed using the Hosmer–Lemeshow goodness-of-fit test. Analyses were conducted in STATA 14^®^ (StataCorp^®^, College Station, TX, USA).

### 4.2. Recruitment of Human Participants and Categorisation of Brucella Infection Evolution

Each herd had a different owner or herd manager, and there was no movement of farm workers between herds included in this study. However, veterinary officials are routinely exposed to more than one herd irrespective of disease status of that herd.

All farm workers (*n* = 150) on case farms (*n* = 30), a subset of farm workers (*n* = 53) on control farms (*n* = 11) and veterinary officials (*n* = 27) servicing all three state vet areas were sampled for testing. On farms where farm workers were tested, farm managers or owners of herds were administered a structured herd management questionnaire face-to-face. The questionnaire collected data on herd management factors, and cattle herd and human symptoms of brucellosis detected as abnormal by the farmer in the year before the last herd test result. The same questionnaire was administered telephonically to the remaining herd managers of the control farms where no testing of farm workers took place.

The seroprevalence study on farm workers was conducted on farm sites between March and November 2016. A multidisciplinary team comprising a veterinarian, medical doctor and animal health technician visited each farm. The animal health technician served as the translator and was pre-trained in administering the questionnaire. The veterinarian administered the herd management questionnaire to the farm manager, while the medical doctor collected blood samples from the study participants. Veterinary officials were sampled at the veterinary offices on appointed days for each state vet area. Five millilitres of blood from each participant was drawn into two tubes: (1) clot activator without serum separation (dry tubes) and (2) EDTA anticoagulant tube blood samples were transported on ice to the National Institute for Communicable Diseases, Centre for Emerging Zoonotic and Parasitic Diseases Unit, by the medical doctor for further processing, immediately following the farm visit. At the unit, samples were refrigerated (2–8 °C) until they were processed. Processing was performed within a week of receipt.

Human samples were tested using commercially available kits for the RBT^®^, IgM Enzyme Linked Immunosorbent Assay^®^ (ELISA), IgG ELISA^®^ [70,71,72] and BrucellaCap^®^t immunocapture serological test [73] according to the manufacturers’ instructions [74,75] and results were interpreted according to the kit guidelines.

Subjects with insufficient blood for the RBT^®^ (*n* = 2) were excluded from the analysis. All samples were tested with the RBT^®^, IgG ELISA^®^ and BrucellaCapt^®^. Samples that were seropositive on the ELISA IgG^®^ were tested further using the IgM ELISA^®^. Samples seronegative on the IgG ELISA^®^, but seropositive using the RBT^®^, were also subjected to an IgM ELISA^®^. This selective testing of samples using the IgM ELISA^®^ was due a limited budget. The purpose was to detect the presence or absence of *Brucella* IgM antibodies in these selected samples to better understand the evolutionary stage of infection in the farm workers and veterinary officials. Stages of infection were considered along a continuum from a very short evolution of infection (IgM seropositive, IgG seronegative), reported to last approximately a week after exposure/inoculation with *Brucella* spp. [76], to a long evolution of infection (IgM seronegative, IgG seropositive, possible presence of blocking or non-agglutinating antibodies). As such, each seropositive person fell into one of five mutually exclusive groups depending on the outcome of a combination of tests: (i) RBT^®^ positive and IgM ELISA^®^ positive and IgG ELISA^®^ negative (indicative of a very short evolution infection), (ii) RBT^®^ negative and IgM ELISA^®^ positive and IgG ELISA^®^ positive, (iii) RBT^®^ positive and IgM ELISA^®^ positive and IgG ELISA^®^ positive (indicative of a short evolution infection), (iv) RBT^®^ positive and IgM ELISA^®^ negative and IgG ELISA^®^ positive (indicative of a long evolution infection), and (v) RBT^®^ negative and IgM ELISA^®^ negative and IgG ELISA^®^ positive (indicative of inactive or resolved infection). Seropositive reactors on the BrucellaCapt test were allocated to the group defined by the outcomes of the RBT^®^, IgM ELISA^®^ and IgG ELISA^®^.

Subjects with test results for the IgG ELISA^®^ that were classed as equivocal (*n* = 3) were removed from the analysis. Titres were determined using the BrucellaCapt^®^ test. A titre of greater or equal to 1:320, was considered positive.

Questionnaire responses and test results from human participants were captured into an ACCESS 2013^®^ (Microsoft suite 2013) relational database, using a unique herd identifier to link test results from farm workers to the herd they were in contact with. The farm managers’ questionnaire response shared this unique number.

## 5. Conclusions

To our knowledge, this study was the first transdisciplinary epidemiological field study of bovine brucellosis at the human–cattle farm interface to augment an existing government bovine brucellosis control program. We found evidence of cattle handler exposure to *Brucella* on cattle farms participating in the provincial veterinary services’ bovine brucellosis control programme as well as significant herd risk factors and symptoms.

Variation of seroprevalence amongst cattle handlers according to serological test is consistent with the picture in a brucellosis endemic area. This study suggests the possibility of undetected and untreated cases of brucellosis amongst cattle handlers, including veterinary officials, in the province. Therefore, as suggested by Mantur (2006) for a brucellosis endemic area, we recommend that medical practitioners routinely screen farm workers and family members, and veterinary officials exposed to cattle herds for early detection of infection with *Brucella* using serial dilutions of the RBT test as recommended by Diaz et. al., (2011). In addition, ongoing training to cattle handlers is recommended to increase awareness of the zoonotic occupational risk of brucellosis as well as human symptoms of the disease. Further investigation into the health-seeking behaviour in response to brucellosis-like symptoms amongst RBT and BrucellaCapt seropositive cattle handlers is needed to rule out undetected chronic or relapsing brucellosis. However, commercial screening tests recommended cut-offs need to be adjusted to differentiate between clinical disease and asymptomatic infection in the province.

Interpreted as a whole, findings from this study corroborate a complex “One Health” model of human, cattle and socio-political interrelatedness with respect to bovine brucellosis at the human–cattle farm interface. We therefore recommend a similar “One Health” approach to integrate transdisciplinary public and private resources for further investigation of, and response to, the clinical significance of seropositive cattle handlers and further study on the frequency and distribution of *Brucella* spp. in the region [77] to mitigate the identified herd risk factors and to calculate the economic and socio-economic impacts of bovine brucellosis on farms in Gauteng, South Africa, Sub-Saharan Africa and in other low- to middle-income regions of the world.

## Figures and Tables

**Table 1:** Brucella seroprevalence amongst farm workers on case and control farms and veterinary officials according to different serological tests, Gauteng, 2016.

Serological Test	Farm Workers on Control Farms		Farm Workers on Case Farms		Veterinary Officials		Total	
	(*n* = 53)		(*n* = 150)		(*n* = 27)		(*n* = 230)	
	Seropositive	%	Seropositive	%	Seropositive	%	Total	%
RBT^®^	2	3.8	13	8.7	8	29.6	23	10.1
IgG ELISA^®^	3	5.7	25	16.7	20	74.1	48	20.9
BrucellaCapt^®^	1	1.9	6	4.0	8	29.6	15	6.5

**Table 2:** Brucella seropositivity among farm workers and veterinary officials (*n* = 230) on cattle farms in Gauteng, according to combinations of serological tests to indicate prevalence across the evolution of infection.

	Combination of Serological Test Results	Farm Workers on Control Farms		Farm Workers on Case Farms		Veterinary Officials		Total	
		(*n* = 53)		(*n* = 150)		(*n* = 27)		(*n* = 230)	
		Seropositive	%	Seropositive	%	Seropositive	%	Total	%
**Evolution of Infection**	(i) RBT+ IgM+ IgG-	1	1.9	1	0.7	0	0	2	0.9
	(ii) RBT- IgM+ IgG+	2	3.8	2	1.3	3	11.1	7	3
	(iii a) RBT+ IgM+ IgG+	1	1.9	9	6	3	11.1	13	5.7
	(iii b) RBT+ IgM+ IgG+ BrucellaCapt +	1	1.9	5	3.3	3	11.1	9	3.9
	(iv a) RBT+ IgM- IgG+	0	0	3	2	5	18.5	8	3.5
	(iv b) RBT+ IgM- IgG+ BrucellaCapt +	0	0	0	0	5	18.5	5	2.2
	(v a) RBT- IgM- IgG+	0	0	11	7.3	9	33.3	20	8.7
	(v b) RBT- IgM- IgG+ BrucellaCapt +	0	0	1	0.7	0	0	1	0.4

**Table 3:** Univariate and multivariable analysis of herd management factors associated with herd *Brucella* infection status in cattle herds in Gauteng, 2014–2016.

Variable and Level	Univariate Analysis					Multivariable Analysis		
	Case Farms (*n* = 30)		Control Farms (*n* = 103)		*p*-Value			
	*n*	%	*n*	%		Odds Ratio	95% CI	*p*-Value
Government Sponsored					0.009			
No	18	60	87	84.5		1	–	−
Yes	12	40	16	15.5		4.0	1.4–11.3	0.009
State Veterinary Area					0.751			
Pretoria (base)	15	50	50	48.5				
Randfontein	6	20	27	26.2				
Germiston	9	30	26	25.2				
Herd Type					0.021			
Dairy (base)	2	6.7	32	31.1		1	–	–
Beef	23	76.7	54	52.4		7.9	1.4–44.9	0.020
Mixed	5	16.7	17	16.5		4.0	0.5–30.6	0.187
Presence of antelope					< 0.001			
No	23	76.7	101	98.1		1	–	–
Yes	7	23.3	2	1.9		29.4	4.0–218.2	0.001
*Brucella* vaccination (S19/RB51)					0.404			
Yes	27	90	84	81.6				
No	3	10	19	18.4				
Open Herd					0.032	1	–	–
No	6	20	44	42.7		3.3	1.1–10.4	0.038
Yes	24	80	59	57.3				
Herd Size (Quartiles)					0.093			
3–37 (base)	6	20	24	23.3				
38–88	3	10	28	27.2				
89–200	13	43.3	24	23.3				
>200	8	26.7	27	26.2				
Calving					0.3304			
Separated	7	25	25	35.2				
Together	21	75	46	64.8				
Bull					0.275			
Use bull from own herd	21	70	77	74.8				
Use bull from another herd	5	16.7	6	5.8				
Use bull & AI	3	10	14	13.6				
Use AI only	1	3.3	6	5.8				
Farm fenced in					0.818			
No	9	30	28	27.2				
Yes	21	70	75	72.8				
Handling facilities					0.419			
None (base)	2	6.7	7	6.8				
Poor	8	26.7	23	22.3				
Good	16	53.3	44	42.7				
Excellent	4	13.3	29	28.2				
Brucellosis in neighbouring herds					0.046			
No	25	83.3	98	95.1				
Yes	5	16.7	5	4.9				

**Table 4:** Univariate analysis of farmer-reported cattle and human symptoms associated with herd *Brucella* infection status herds in Gauteng 2014–2016.

Variable and Level	Univariate Analysis				
	Case Farms (*n* = 30)		Control Farms (*n* = 103)		*p*-Value
	*n*	%	*n*	%	
Abortions					<0.001
No	13	43.3	82	79.6	
Yes	17	56.7	21	20.4	
Retained placentas					0.156
No	23	76.7	90	87.4	
Yes	7	23.3	13	12.6	
Weak calves in herd					<0.001
No	17	56.7	94	91.2	
Yes	13	43.3	9	8.7	
Reduction in number of calves born					<0.001
No	22	73.3	99	96.1	
Yes	8	26.7	4	3.9	
Reduction in milk yield					1
No	27	90	92	89.3	
Yes	3	10	11	10.7	
Reduction in conception rate		0			0.046
No	25	83.3	98	95.1	
Yes	5	16.7	5	4.9	
Hygromas in cattle					0.011
No	27	90	103	100	
Yes	3	10	0	0	
Farmer reported brucellosis-like symptoms in farm workers or self					0.006
No	11	36.7	68	66.0	
Yes	19	63.3	35	34.0	

**Table 5:** Description of selected herd management risk factors.

Risk Factors	Description
Brucella vaccination (RB51)	The herd has a history of vaccination preceding the herd test result
Open Herd	Cattle (heifers, cows or bulls) are bought in or are a part of a communal herd (multiple owners) as opposed to a herd that uses its own replacement heifers, bulls or only AI.
Government Sponsored	Farmers have received a grant to farm or access to purchase cattle, buy or rent land as part of governmental redress of apartheid policies to support previously disadvantaged persons to farm.
Herd Type	Dairy (Fresian/Jersey)
	Beef (Bonsmara/Brahman/Nguni)
	Mixed (Beef breed/s and dairy breed/s)
Handling Facilities	An assessment of handling facility quality by the farmer/manager
Brucellosis in Neighbouring Herds	Neighbouring farmers reporting the brucellosis status of their herds to the manager/owner being interviewed.

## Data Availability

Data are available on request from the Gauteng Department of Agriculture and Rural Development.
